# Functional Disability and Symptomatic Slow-Acting Drugs for Osteoarthritis in Adults with Periodontitis

**DOI:** 10.3390/healthcare11050770

**Published:** 2023-03-06

**Authors:** Nik-Madihah Nik-Azis, Nurulhuda Mohd, Badiah Baharin, Fazalina Mohd Fadzilah, Nor Hazla Mohamed Haflah, Mohd Shahrir Mohamed Said

**Affiliations:** 1 Department of Restorative Dentistry, Faculty of Dentistry, Universiti Kebangsaaan Malaysia, Jalan Raja Muda Abdul Aziz, Kuala Lumpur 50300, Malaysia; 2 Radiology Department, Sunway Medical Centre, Bandar Sunway, Selangor 47500, Malaysia; 3 Department of Orthopaedics and Traumatology, Faculty of Medicine, Hospital Canselor Tuanku Mukhriz, Jalan Yaacob Latif, Bandar Tun Razak Cheras, Kuala Lumpur 56000, Malaysia; 4 Faculty of Medicine, Hospital Canselor Tuanku Mukhriz, Jalan Yaacob Latif, Bandar Tun Razak Cheras, Kuala Lumpur 56000, Malaysia

**Keywords:** periodontal disease, health assessment questionnaire, functional disability, symptomatic slow-acting drugs in osteoarthritis

## Abstract

Osteoarthritis (OA) patients have decreased functional ability and restricted access to healthcare facilities and are on a spectrum of medications. These can impact their oral health. This study aims to investigate the association between periodontal disease and OA disease parameters, specifically the functional disability and the medications taken. This was a cross-sectional study on OA participants recruited from the Hospital Canselor Tuanku Mukhriz. Periodontal health parameters were obtained from an oral examination of the participants. A Health Assessment Questionnaire (HAQ) was administered to ascertain the functional status of the participants. Out of the 130 participants recruited, 71 (54.6%) had periodontitis. There was a correlation between the teeth count with OA severity, where participants with a greater Kellgren–Lawrence score had less teeth (rs = 0.204, *p* = 0.025). Participants with a greater degree of functional limitation also had less teeth (rs = −0.181, *p* = 0.039) and a higher clinical attachment loss (rs = 0.239, *p* = 0.006). There were no associations found between the symptomatic slow-acting drugs in OA and periodontal health parameters. In conclusion, there was a high proportion of periodontitis in patients with OA. Functional disability was associated with measures of periodontal health. It is suggested that clinicians treating OA patients consider the need for a referral for dental care when managing this group of patients.

## 1. Introduction

Osteoarthritis (OA) is traditionally described as a ‘wear and tear’, mechanically driven disease, that is, a consequence of the aging process affecting mostly older adults [1]. It is a disorder involving movable joints, characterized by cell stress and extracellular matrix degradation initiated by micro- and macro-injury. This process stimulates maladaptive repair responses, including pro-inflammatory pathways of the innate immunity [2,3]. More contemporary views of OA argued that the inflammation contributes to OA synovitis and its pathology [4]. OA is now viewed as a complex disease, with inflammatory mediators released by cartilage, bone and synovium and its progression governed by a set of multifactorial components [5]. The role of inflammation in this new paradigm highlights inflammatory signals, including cytokines, surface-expressed pattern recognition receptors, complement factors, pathogen-associated molecular patterns and damage-associated molecular patterns that can lead to the degradation of the cartilage matrix in OA [5].

Periodontal disease (PD) is a disease that affects the supporting structures of the teeth. It is characterized by microbially associated, host-mediated inflammation that results in loss of periodontal attachment [6]. Periodontitis is an immune-inflammatory infection that can cause low-grade systemic inflammation, which may influence the development of systemic comorbidities. The periodontitis-associated systemic inflammation can cause hematogenous dissemination of periodontal bacteria and inflammatory mediators from periodontal tissues to the bloodstream [7,8]. This can later spread to other parts of the body. Despite the similarities between the role of inflammation in the pathogenesis of OA and PD, limited studies have investigated the association between the two diseases and its related factors.

Chung and co-workers studied the data of 7969 adults from the Korea National Health and Nutrition Examination Survey (KNHANES) during 2010–2014. They reported that severe OA and periodontitis were associated with each other in a subgroup analysis involving female patients [9]. Kim et al. also reported that periodontitis was associated with the presence and severity of radiographic knee OA in their Korean nationwide study, also using data from the KNHANES [10]. Other studies on periodontal disease and OA were case-control studies where OA participants were recruited as controls. These studies include those from United States veterans, reporting 26.4% of OA participants with periodontitis [11], and Vietnam, where 28% of OA participants had periodontitis [12].

OA can involve the joints of the upper extremities, causing functional limitations of the hand. In a study on OA participants, radiographic changes due to OA were found to be associated with reduced grip and pinch strength [13]. This can limit the ability of the participants to utilize oral hygiene aids and remove plaque efficiently [14]. Apart from that, patients with OA can have mobility limitations that affect their ability to access the dental clinic for routine dental care [14,15,16]. The accessibility of dental services and level of patient care, as well as transport availability, were suggested as factors that affect the use of dental services by patients with arthritis [16].

Oral medications used to treat OA can broadly be categorized into analgesics, including paracetamol; non-steroidal anti-inflammatory drugs (NSAIDs); and symptomatic slow acting drugs for OA (SYSADOAs), including glucosamine, chondroitin sulphate, diacerein and avocado and soybean unsaponifiable (ASU). There is evidence that NSAIDs can cause reduced bleeding and a reduction in the rate of bone resorption in observational and intervention studies [17,18]. Diacerein has been hypothesized to be a potential therapeutic drug for periodontitis due to its anti-inflammatory activities that selectively inhibits signal transduction affecting cytokine profiles and ameliorating disease breakdown [19]. In an animal study investigating the effects of diacerein in the management of ligature-induced experimental periodontitis in rats, significant decreases in the IL-1ß level in the test group suggest that diacerein may play a therapeutic role as a potent anti-inflammatory drug in the management of periodontitis [20].

ASU is routinely prescribed in OA and has been suggested as an adjunct for the treatment of periodontitis. ASU added to gingival fibroblasts in culture showed the potential to prevent the deleterious effects of IL-1ß in periodontitis [21]. Animal studies in rats also showed that ASU prescribed as an adjunct to conventional mechanical debridement has subtle beneficial effects on periodontal repair [22]. However, a clinical study found that ASU did not have a favorable effect in the treatment of chronic periodontitis [23].

Current evidence shows that glucosamine exhibits anti-inflammatory effects by reducing the levels of pro-inflammatory factors, such as tumor necrosis factor-alpha, interleukin-1 and interleukin-6 [24]. There is, however, limited data on the effects of glucosamine and chondroitin sulphate on periodontitis. A randomized controlled trial investigated the effects of Arthocare Forte containing chondroitin (400 mg) and glucosamine sulphates (500 mg) administered to patients with tooth mobility over a period of 5 years [25]. The participants underwent non-surgical periodontal therapy, and half were treated with Arthocare Forte. They found that the medication speeds up the regenerative capacity and the stability of the periodontium compared to the control group. 

Generally, there is limited evidence on the effects of medications, such as the SYSADOAs and NSAIDs used to treat OA, on the oral health parameters and periodontal disease. Further studies are needed in this area to assist in understanding how these medications affect specific oral health parameters and whether there is potential for these medications to be used in the treatment of periodontitis.

It was hypothesized that: (a) the prevalence and severity of PD is higher in participants with OA compared to the general population; (b) participants with functional limitations have a greater PD severity, and (c) there is a difference in the PD among participants taking the different SYSADOAs. Hence, this study aimed to investigate: (a) the prevalence and severity of PD among patients with OA compared to the general population; (b) the association between PD parameters and OA parameters, including functional limitations, and (c) the differences in PD among participants taking different SYSADOAs. 

## 2. Materials and Methods

**Figure 1:**
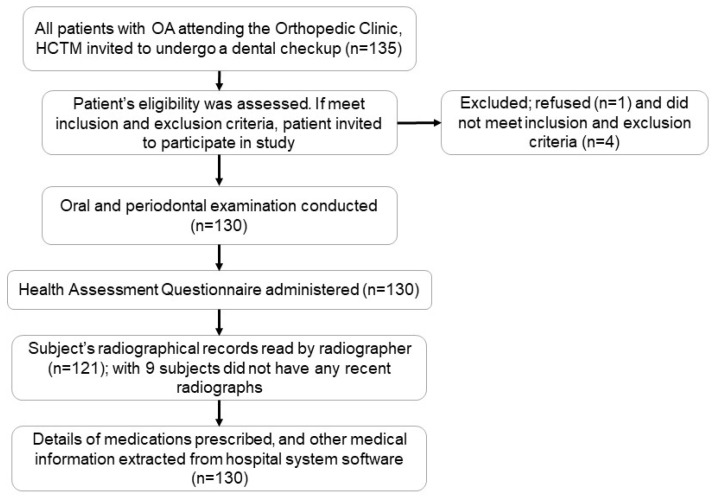
Flowchart of the recruitment and the data collection process, including the number of participants for every step.

The participants were recruited from the Osteoarthritis Clinic under the Orthopaedic Department in Hospital Canselor Tuanku Mukhriz, Malaysia, using convenience sampling. Consecutive patients attending the OA Clinic who met the study criteria were invited to participate in the study. The flowchart of the recruitment process is shown in Figure 1.

The inclusion criteria were: (i) OA patients, as confirmed by the ACR Classification, (ii) above the age of 18 years old, (iii) dentate and (iv) able to give verbal and written consent. The exclusion criteria were: (1) patients who were completely unable to read, write or understand the Malay or English Language; (2) coexistence of other autoimmune diseases; (3) uncontrolled systemic disease or malignancy; (4) patients who were pregnant or planning to become pregnant; (5) any current or previous history of periodontal treatment, including root surface debridement/ periodontal surgery; and (6) any previous or current use of phenytoin or cyclosporin.

This study was conducted as part of research assessing oral health in patients with joint diseases [26]. Ethical approval for the study was obtained from the Ethical Board of the Universiti Kebangsaan Malaysia (UKM/PPI/111/8/JEP-2017-553), and the study conformed to the provisions of the Declaration of Helsinki (as revised in Brazil 2013). All participants of the study gave informed consent, and the anonymity of the participants has been preserved in the conduct and reporting of this study.

### 2.1. Oral Examination

The assessment of the oral and periodontal health used parameters such as the number of remaining teeth, the Plaque Index [27], the Gingival Index [28], the probing pocket depth (periodontitis) and clinical attachment loss (CAL). For the Plaque Index, the original O’Leary protocol used a disclosing solution (such as Bismarck Brown Iodine Stain). However, the disclosing solution was not used in our study. A dichotomous scoring system of (present/absent) was used. Six sites per tooth, namely, the mesiobuccal, midbuccal, distobuccal, mesiopalatal, midpalatal and distopalatal sites, were assessed for both the Plaque Index and the Gingival Index. The periodontal charting was carried out using the University of North Carolina-15 (UNC15) probe. The clinical attachment loss (CAL) was then calculated as a sum of the pocket depth and recession.

Diagnosis of periodontitis was made based on the criteria as outlined by Papapanou et al. [29]. They are as follows: (1) interdental CAL is detectable at ≥2 non-adjacent teeth, or (2) buccal or oral CAL ≥3 mm with pocketing ≥3 mm is detectable at ≥2 teeth, but the observed CAL cannot be ascribed to non-periodontitis-related causes. The periodontal findings were also later recoded to the Community Periodontal Index (CPI) [30] to allow for comparison with the national data available for Malaysia [31]. Oral examination was conducted by a single examiner (NMNA). Prior to the initiation of the study, NMNA was calibrated against a gold-standard periodontist (NM) to ensure inter- and intra-examiner reliability.

Patients who were diagnosed with any dental disease were given a follow-up appointment or advised to seek care at any dental clinic if they were not able to attend for the appointment given.

### 2.2. Measures of Functional Limitations

The Malaysian version of the Health Assessment Questionnaire (HAQ) [32] was administered to ascertain the functional status of the subject. The authors have permission to use this instrument from the copyright holders. The HAQ is a questionnaire with eight sections (Dressing and Grooming, Arising, Eating, Walking, Hygiene, Reach, Grip and Activities) that is validated to assess the functional limitations in participants with musculoskeletal diseases. The HAQ also included vertical, 100 mm, patient global and pain visual analogue scales. Administration of the questionnaire was done by a single researcher prior to collection of the oral health and OA parameters to minimize risk of bias.

### 2.3. Osteoarthritis Parameters

The patient’s medical notes were accessed to extract information regarding the disease duration, type of OA and prescribed medications. The prescription of any medications were noted, and the type of SYSADOAs and painkiller taken were specifically extracted from the hospital patient system.

The participants’ radiographs were read by a musculoskeletal radiologist (FMF) to determine the Kellgren–Lawrence score [33]. Prior to reading the radiographs, FMF was calibrated to another independent musculoskeletal radiologist. The radiographs were each assigned a grade from 0 to 4. The grades correlated to the increasing severity of OA, with Grade 0 signifying no presence of OA and Grade 4 signifying severe OA. For participants suffering a combination of OA where more than one type of joint is affected, the Kellgren–Lawrence score was taken from the joint that was most severely affected. Examiner FMF was blinded to the clinical findings of the participants. The extracted information was checked to ensure that it was dated no longer than three months in duration from the clinical examination.

### 2.4. Statistical Analysis

Statistical analysis of variables was performed with IBM SPSS version 19.0 (IBM Co., Armonk, NY, USA). Univariate comparisons were made using the Chi-squared test. The correlations between periodontal indices and OA disease characteristics were analyzed by Pearson or Spearman correlation coefficients to test for the association between the variables. The Mann–Whitney U test was applied for analysis of independent nonparametric variables. All *p* values are two-sided, and *p* values less than 0.05 were considered statistically significant.

## 3. Results

### 3.1. Demographic Data

The flowchart of the steps of subject recruitment and data collection is reported in Figure 1. The demographic characteristics and periodontitis diagnosis of the study participants are shown in Table 1. The participants were mostly older adults with a mean age of 61.5 (±9.3). Out of the 130 participants with OA, 98 (75.4%) had knee OA, 7 (5.4%) had hip OA and 6 (4.6%) had hand OA, while 19 (14.6%) had a combination type of OA.

### 3.2. Proportion of Osteoarthritis Participants with Periodontitis

**Figure 2:**
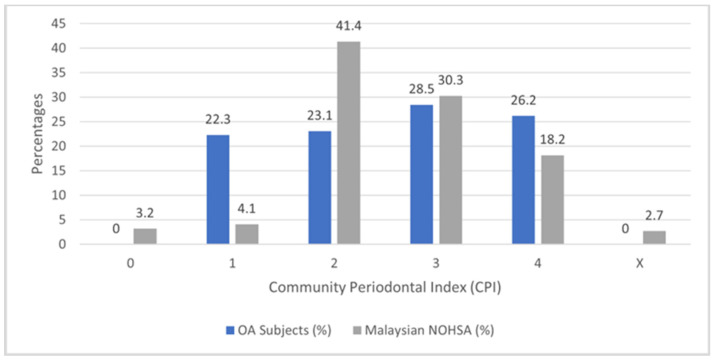
Community Periodontal Index (CPI) of osteoarthritis subjects compared to the data taken from the National Oral Health Survey, 2010.

From the 130 participants recruited, 71 (54.6%) had periodontitis. Generally, the OA participants had a higher proportion of periodontitis compared to the Malaysian NOHSA findings of 48.5% [34]. The severity of periodontitis suffered by the OA participants was also greater, with more participants having a CPI of 4 compared to the NOHSA findings. This is shown in Figure 2.

### 3.3. Osteoarthritis Parameters with Periodontal Health Parameters

The mean ± standard deviation for the OA parameters measured are as follows: OA disease duration: 6.08 ± 6.40; Kellgren–Lawrence Score: 2.95 ± 1.01; HAQ Score: 0.40 ± 0.43; Pain Score: 5.04 ± 2.96; and Global Health Score 7.37 ± 1.80. The distribution of the PD parameters and their correlation with the OA parameters are shown in Table 2. There was a correlation using the Spearman Rho test between the Kellgren–Lawrence score of the participants with teeth count, where participants with a greater Kellgren–Lawrence score had less teeth (rs = 0.204, *p* = 0.025, two-tailed, N = 121). There was no other correlation between any parameters of PD (plaque index, gingival index, probing pocket depth and clinical attachment loss) with the Kellgren–Lawrence score.

The overall HAQ score was correlated with the teeth count (rs = −0.181, *p* = 0.039), average PPD (rs = 0.209, *p* = 0.017) and CAL (rs = 0.239, *p* = 0.006). Participants with a greater degree of functional limitation, as shown by the HAQ score, had worst periodontal health, with less teeth, a higher PPD and a higher CAL. The global health score was inversely correlated with the plaque index (rs = −0.178, *p* = 0.043), where participants with a higher global health score had less plaque.

The specific sections of the HAQ were further investigated against the oral health parameters to ascertain which type of functional limitations was correlated with which oral health parameter. Out of the eight HAQ sections, only ‘Arising’, ‘Eating’ and ‘Walking’ were correlated with specific oral health parameters. Participants reporting a limitation in ‘Arising’ and ‘Eating’ had deeper CAL (rs = 0.206, *p* = 0.019; rs = 0.211 *p* = 0.016) and PPD (rs = 0.245, *p* = 0.005; rs = 0.278 *p* = 0.001). Both CAL and PPD are parameters related to periodontal health, indicating that participants with a limitation in ‘Arising’ and ‘Eating’ had worst periodontal health. A limitation in ‘Walking’ was correlated with all 5 oral health parameters investigated, namely, teeth count (rs = −0.233, *p* = 0.008), plaque index (rs = 0.195, *p* = 0.026), gingival index (rs = 0.177, *p* = 0.045), average PPD (rs = 0.209, *p* = 0.017) and average CAL (rs = 0.265, *p* = 0.002). All five oral health parameters are measures of the overall oral health, indicating that participants with walking limitations have worst oral and periodontal health.

### 3.4. Medications and Periodontal Health Parameters

Table 3 shows the type of SYSADOAs taken by the participants. Most of the participants were not taking any SYSADOAs (53.8%), while those who were taking SYSADOAs were mostly prescribed either Glucosamine (13.1%) or Piascledine (27.7%). There was no difference in the periodontal health parameters between participants taking different SYSADOA medications and when compared to those not on any medications. There was also no difference in the oral health parameters between the participants taking NSAIDs and those who were not.

## 4. Discussion

Osteoarthritis is a complex condition affecting the whole joint, where an association with systemic inflammation could also be present [35]. OA is now viewed as a complex biological response connecting biomechanics, inflammation and the immune system, rather than a purely mechanical disease due to the wear and tear of the cartilage in the joints [5]. The prevalence of periodontitis among patients with OA in this study is higher at 54.6% when compared to the national average of 48.5% [31]. Other reports on the prevalence of periodontitis in OA participants reported a relatively lower prevalence of 39.4% [10], 26% [36], 26.4% [11] and 28% [12]. This increased prevalence can be attributed to the limitations in function, as well as the increase in the overall systemic inflammation experienced by participants with OA. Other factors include the poorer general health of the subjects, as well as the older age of the participants. 

The results showed that participants who reported functional limitations in the HAQ had a more severe form of periodontitis with less teeth, a higher PPD and a greater CAL. Participants with a limitation in ‘Arising’ and ‘Walking’ had the worst periodontal health. This can be attributed to the mobility limitations that affect the OA participants’ ability to access the dental clinic for routine dental care [14,15,16]. Patients with arthritis can be limited in their access to dental services and may also have transportation restrictions, which are factors affecting the use of dental services by this group of patients [16]. In view of the increased severity of periodontal disease in this group of patients, dental services should be made more accessible, such as by offering home visits and having the location of the dental clinics be more accessible, such as on the ground floor or with elevators or ramps to better facilitate the dental visits of these OA patients. Dental healthcare personnel can also visit musculoskeletal clinics to provide care at regular intervals.

The correlation between a limitation in ‘Eating’ and the severity of periodontitis, as measured by CAL and PPD, suggests that OA patients with nutrition issues may be more predisposed to PD. Bone formation, healing of the periodontium and periodontal regeneration are affected by various vitamins, minerals and trace elements, such as vitamin C, vitamin D, magnesium and calcium [37,38]. It is possible that the limitation in eating in this population contributes to the increased prevalence and severity of PD in patients with OA [37,38,39]. The assessment of diet during dental visits can allow the clinician to better address the factors related to the periodontal disease among patients with OA. Referral to a dietitian can also be a strategy for the improvement of both the oral health and the general health of this group of patients.

Restrictions in the ability to grip objects can lead to poorer oral hygiene, worsening of the periodontal health clinical parameters and poorer oral health. However, there was no correlation between limitations in ‘Grip’ with any oral health parameters in this study. This can be attributed to the relatively low number of subjects with hand OA, which was 14.6%. The tool used to assess functional limitations in this study, which was the HAQ, were also not designed to specifically assess hand function. This can explain the lack of correlation between ‘Grip’ limitations and the oral health parameters.

This study found an association between the severity of OA and the number of teeth in the mouth, where participants with a more severe form of OA had less teeth. The association can be an indicator that the two diseases may be related. However, it is also possible that the association is due to the participant’s age, where the mean age of the participants in this study is 61.5 years. With increasing age, participants will tend to have a greater severity of OA and will also have less teeth, as the estimate of tooth loss is 0.2 teeth per year [40]. Tooth loss can lead to the reduced nutritional value of the food intake, as well as a reduction in social participation that can eventually lead to social isolation and depression. Preventive measures to limit tooth loss, as well as the provision of a prothesis to replace teeth, can be invaluable for these patients. 

Medications taken for OA termed SYSADOAs have been reported to have beneficial effects on the periodontium. Diacerein has anti-inflammatory activities that affects the cytokine profiles [19], while ASU cultured with gingival fibroblasts showed potential to prevent the deleterious effects of IL-1ß in periodontitis [21], and supplements containing chondroitin and glucosamine sulphate have been reported to speed up the regenerative capacity and stability of the periodontium [25]. However, there was no significant difference of the periodontal health parameters between the participants taking the different medications for OA in this study. This can be due to the cross-sectional nature of this study, where the longitudinal effects of these medications cannot be accurately detected, as well as the relatively small numbers of participants taking the different types of SYSADOAs. In this group of participants, not all of the SYSADOAs are available in the clinics, and some of them had to be purchased privately. This may impact the type of medication taken for the OA, depending on the socio-economic background of the participants.

The limitations of this study include the methods of measurement for the functional disability. This study utilized a self-reported assessment of function and disability as assessed by the HAQ. Other methods of assessment may be questionnaires that specifically assess the hand function, such as the Functional Index for Hand Arthropathies (FIHOA), or a performance-based test, such as the Arthritis Hand Function Test (AHFT). A more objective measurement of hand function will give a better picture of the ability of the subjects to perform regular oral hygiene care. The older age of the participants in this study may also be a confounding factor affecting the periodontal health, as well as the functional limitations, of the participants. Other limitations are that factors such as the duration, compliance and dose of the medications taken were not analyzed. These may play a role in the relationship between the medications and PD and should ideally be analyzed in the future. 

This study is one of very few studies reporting on the proportion of OA subjects with periodontitis. The findings of this study can be useful for dental practitioners and policymakers to have a better understanding of the factors and clinical parameters related to the oral and periodontal health of OA patients. It can also be used to design a more rigorous study investigating factors linking OA and periodontitis in the form of cohort studies or randomized controlled trials. 

## 5. Conclusions

There is a high proportion of periodontitis among patients with musculoskeletal disease, namely, OA. Functional disability among OA patients was associated with clinical measures of periodontal health. Within the limitations of this study, SYSADOAs taken by the participants were not associated with the diagnosis, stage, grade and periodontal health parameters in OA participants. It is suggested that clinicians treating OA patients consider the need for a referral for dental care when managing this group of patients. 

## Figures and Tables

**Table 1:** Sociodemographic characteristics and the periodontal diagnosis of the study participants.

Sociodemographic Characteristics (N = 130)		
Gender (n [%])	Female	106 (81.54)
	Male	24 (18.44)
Race (n [%])	Malay	74 (56.92)
	Chinese	40 (30.77)
	Indian	14 (10.77)
	Other	2 (1.54)
Age (mean ± SD)	Age	61.5 (± 9.3)
Education (n [%])	Primary	48 (36.92)
	Secondary	53 (40.77)
	Tertiary	29 (22.31)
BMI (mean ± SD)	Kg/m^2^	28.4 (± 5.1)
Smoking (n [%])	No	121 (93.08)
	Past	4 (3.08)
	Current	5 (3.85)
Diabetes (n [%])	Yes	44 (33.85)
	No	86 (66.15)
**Periodontal Diagnosis, Stage and Grade**		
Diagnosis (n = 130)		n (%)
Healthy		34 (26.2)
Gingivitis		25 (19.2)
Periodontitis		71 (54.6)
Stage of Periodontitis (n = 71)		
Stage 1		17 (13.1)
Stage 2		19 (14.6)
Stage 3		21 (16.2)
Stage 4		14 (10.8)
Grade of Periodontitis (n = 71)		
Grade A		6 (8.5)
Grade B		58 (81.7)
Grade C		7 (9.9)

**Table 2:** PD parameters and their correlation with the OA parameters.

	Teeth Count	Plaque Index	Gingival Index	Average PPD	Average CAL
**mean ± SD**	18.93 ± 7.63	45.45 ± 32.12	40.27 ± 31.90	12.56 ± 5.86	1.34 ± 1.39
Correlation between PD and OA parameters					
OA Disease Duration; r_s_ (*p*-value)	−0.173 (0.048 *)	0.119 (0.177)	0.124 (0.162)	0.060 (0.495)	0.039 (0.660)
Kellgren–Lawrence Score; r_s_ (*p*-value)	−0.204 (0.025 *)	0.063 (0.492)	0.071 (0.438)	0.006 (0.951)	0.066 (0.471)
HAQ; r_s_ (*p* value)	−0.181 (0.039 *)	0.143 (0.105)	0.153 (0.083)	0.209 (0.017 *)	0.239 (0.006 *)
Pain Score; r_s_ (*p* value)	0.041 (0.643)	0.047 (0.598)	−0.074 (0.408)	−0.032 (0.714)	−0.047 (0.597)
Global Health Score; r_s_ (*p* value)	0.125 (0.158)	−0.178 (0.043 *)	0.143 (0.105)	−0.150 (0.088)	−0.164 (0.062)

* *p*-value < 0.05; PPD—periodontal probing depth; CAL—clinical attachment loss; HAQ—health assessment questionnaire.

**Table 3:** The type of Symptomatic Slow Acting Drugs for Osteoarthritis (SYSADOAs) prescribed and the Periodontal Health parameters according to the SYSADOAs prescribed.

Medication Type	Glucosamine	Diascerine	Piascledine	Combination	No	*p*-Value
N (%)	17 (13.1)	3 (2.3)	36 (27.7)	4 (3.1)	70 (53.8)	
Periodontal Diagnosis						
Healthy; n (%)	5 (29.4)	1 (33.3)	12 (33.3)	1 (25.0)	34 (26.2)	0.667
Gingivitis; n (%)	4 (23.5)	0	5 (13.9)	2 (50.0)	25 (19.2)	
Periodontitis; n (%)	8 (47.1)	2 (66.7)	19 (52.8)	1 (25.0)	71 (54.6)	
Oral Health Parameters						
Teeth Count; (mean ± SD)	19.35 ± 6.89	19.33 ± 12.50	18.36 ± 8.03	26.50 ± 1.29	18.67 ± 7.54	0.373
Plaque Index; (mean ± SD)	40.88 ± 31.24	53.33 ± 45.17	44.17 ± 33.1	40.00 ± 14.14	47.19 ± 32.65	0.927
Gingival Index; (mean ± SD)	35.94 ± 33.28	51.67 ± 47.52	37.78 ± 34.44	36.25 ± 12.50	42.29 ± 30.89	0.875
Average PPD; (mean ± SD)	1.98 ± 1.25	2.84 ± 2.10	1.88 ± 1.09	1.50 ± 0.42	2.04 ± 1.29	0.660
Average CAL; (mean ± SD)	2.98 ± 2.33	4.51 ± 4.18	3.38 ± 2.13	3.38 ± 2.13	3.37 ± 2.38	0.871

## Data Availability

Data available on request due to restrictions.
